# Mechanisms regulating resistance to inhibitors of topoisomerase II

**DOI:** 10.3389/fphar.2013.00089

**Published:** 2013-08-01

**Authors:** Ram N. Ganapathi, Mahrukh K. Ganapathi

**Affiliations:** Levine Cancer Institute, Carolinas HealthCare System Charlotte, NC, USA

**Keywords:** drug resistance, topoisomerase II, calmodulin inhibitors, topo II phosphorylation, casein kinase I

## Abstract

Inhibitors of topoisomerase II (topo II) are clinically effective in the management of hematological malignancies and solid tumors. The efficacy of anti-tumor drugs targeting topo II is often limited by resistance and studies with *in vitro* cell culture models have provided several insights on potential mechanisms. Multidrug transporters that are involved in the efflux and consequently reduced cytotoxicity of diverse anti-tumor agents suggest that they play an important role in resistance to clinically active drugs. However, in clinical trials, modulating the multidrug-resistant phenotype with agents that inhibit the efflux pump has not had an impact. Since reduced drug accumulation *per se* is insufficient to explain tumor cell resistance to topo II inhibitors several studies have focused on characterizing mechanisms that impact on DNA damage mediated by drugs that target the enzyme. Mammalian topo IIα and topo IIβ isozymes exhibit similar catalytic, but different biologic, activities. Whereas topo IIα is associated with cell division, topo IIβ is involved in differentiation. In addition to site specific mutations that can affect drug-induced topo II-mediated DNA damage, post-translation modification of topo II primarily by phosphorylation can potentially affect enzyme-mediated DNA damage and the downstream cytotoxic response of drugs targeting topo II. Signaling pathways that can affect phosphorylation and changes in intracellular calcium levels/calcium dependent signaling that can regulate site-specific phosphorylation of topoisomerase have an impact on downstream cytotoxic effects of topo II inhibitors. Overall, tumor cell resistance to inhibitors of topo II is a complex process that is orchestrated not only by cellular pharmacokinetics but more importantly by enzymatic alterations that govern the intrinsic drug sensitivity.

## INTRODUCTION

The emergence of drug-resistant tumor cells continues to be a major problem confronting advances in cancer chemotherapy. Resistance to the various classes of anti-tumor agents (Curt et al., 1984) has been suggested to involve reduced drug accumulation and/or retention, conformational changes and/or over production of the target enzyme, and reduced activation and/or increased catabolism of drug. Doxorubicin (DOX) is a clinically effective anti-tumor agent against a spectrum of neoplastic diseases (Carter, 1975; Myers and Chabner, 1990). Although DOX is an inhibitor of topoisomerase II (topo II), multifactorial mechanisms are involved in the cytotoxic response (Siegfried et al., 1985; Louie et al., 1986; Bhushan et al., 1989; Doroshow et al., 1990). Pioneering studies of Kessel et al. (1968) and Biedler and Riehm (1980) established that reduced drug accumulation in tumor cells is a major mechanism involved in resistance to clinically important anti-tumor agents, e.g., anthracyclines and vinca-alkaloids. The overexpression of P-glycoprotein (PGP) in resistant cells, which mediates energy dependent drug efflux across a concentration gradient and is responsible for reduced drug accumulation, was originally described by Ling and Thompson (1974). The cross-resistance to anti-tumor drugs of diverse structure and/or mechanism of action (Endicott and Ling, 1989; Chin et al., 1993) mediated by PGP is now termed multidrug resistance (MDR). MDR in the absence of overexpression of PGP has been demonstrated to be due to the MDR related protein (MRP), which like PGP also belongs to the ATP-binding cassette (ABC) superfamily of membrane proteins (Center, 1993; Cole et al., 1994).

## MODULATION OF DRUG RESISTANCE BY CHEMOSENSITIZERS

Reduced drug accumulation to anti-tumor drugs of diverse structure and mechanism of action has led to the identification of agents that can potentially sensitize tumor cells with the MDR phenotype. The original reports on modulation of MDR by calcium blockers, e.g., verapamil (Slater et al., 1982; Tsuruo et al., 1982) or calmodulin inhibitors, e.g., trifluoperazine (TFP; Tsuruo et al., 1982; Ganapathi and Grabowski, 1983) has been confirmed subsequently by other laboratories in a variety of model systems (Ford and Hait, 1990). Excellent reviews on compounds modulating the MDR phenotype has been published, and sensitization of drug-resistant pre-clinical tumor models *in vivo* has been observed (Tsuruo et al., 1982; Ganapathi et al., 1988; Ford and Hait, 1990). The mechanism of action of the “chemosensitizers” in MDR cells is suggested to involve binding to PGP which results in increased drug accumulation and consequently cytotoxicity. While these chemosensitizers do indeed increase drug accumulation, concentrations of the anti-tumor agent required in resistant cells are significantly higher than those required by the wild-type (sensitive) cells to achieve equivalent cell kill. Based on the promise from pre-clinical studies, clinical trials have evaluated these agents to sensitize drug refractory tumors (Ganapathi et al., 1993a; Lum et al., 1993) but results with a potent inhibitor of PGP indicate that modulation of drug resistance or enhanced clinical activity is not realized (Carlson et al., 2006; Kolitz et al., 2010).

Most studies on modulation of MDR have relied on tumor models with high levels of resistance making it difficult to ascertain whether the resistance to anthracyclines and vinca alkaloids was exclusively due to overexpression of PGP. In addition, the observation that resistance to lipophilic anthracyclines was observed without apparent differences in drug accumulation between sensitive and resistant cells suggested a role for alternate mechanisms of resistance (Ganapathi et al., 1984, 1989). To assess the central role for PGP and probe mechanisms of resistance to DOX we developed progressively DOX-resistant (5- to 40-fold) cell lines of L1210 mouse leukemia and B16-BL6 mouse melanoma (Ganapathi et al., 1987; Ganapathi and Grabowski, 1988). Studies with these progressively resistant tumor models revealed that while the IC50 for DOX alone was higher with increasing resistance (0.25–5 μM), significantly lower concentrations of DOX (0.08–0.7 μM) were required in the presence of a non-cytotoxic concentration (5 μM) of the calmodulin inhibitor TFP to achieve equivalent cell kill (Ganapathi and Grabowski, 1988; Ganapathi et al., 1988). In the progressively DOX-resistant L1210 cells expression of the MDR phenotype was observed only at >10-fold but not at fivefold resistance to DOX and role of PGP in these progressively DOX-resistant cells revealed that: (a) effects of PGP on drug accumulation were correlative with vincristine (VCR) rather than DOX resistance (Ganapathi et al., 1991b, a); and (b) the modulation by TFP of VCR but not DOX cytotoxicity was due to effects on drug accumulation (Ganapathi et al., 1991a, b). Based on the lack of correlation between cellular DOX levels and cytotoxic response, using the progressively DOX-resistant L1210 model system, nuclear levels of DOX were determined following treatment with the IC50 of DOX in the absence or presence of 5 μM TFP (Ganapathi et al., 1991a). Results revealed that significantly higher nuclear levels of DOX were required in the resistant compared to the parental sensitive cells to achieve equivalent cytotoxicity, suggesting that alterations in topo II, a putative target of DOX may be involved (Ganapathi et al., 1991a).

## TOPOISOMERASE II AND DRUG RESISTANCE

The topoisomerases alter DNA topology for the efficient processing of genetic material (Chen and Liu, 1994; Pommier et al., 1994; Watt and Hickson, 1994; Froelich-Ammon and Osheroff, 1995). The two well characterized topoisomerases, topoisomerase I (topo I) and topo II, which are essential for DNA metabolism are also the targets for the clinically effective anti-tumor agents, e.g., analogs of camptothecin (topotecan, irinotecan), DOX, daunorubicin, etoposide (VP-16), or teniposide (Chen and Liu, 1994; Pommier et al., 1994; Watt and Hickson, 1994; Froelich-Ammon and Osheroff, 1995). Eukaryotic topo I catalyzes DNA relaxation via a transient single stranded DNA break while topo II will produce a transient double stranded break for the passage of double stranded DNA segments (Chen and Liu, 1994; Pommier et al., 1994; Watt and Hickson, 1994; Froelich-Ammon and Osheroff, 1995). Anti-cancer drugs which interact with topoisomerases and produce DNA strand breaks, involves the stabilization of a ternary complex with DNA. The single or double stranded break induced by topo II, involves linkage to the 5′-phosphoryl end of the broken DNA, with the 5′ broken end protruding precisely four bases with a double strand break (Chen and Liu, 1994; Pommier et al., 1994; Watt and Hickson, 1994; Froelich-Ammon and Osheroff, 1995). The mechanism of DNA strand breakage induced by topo II inhibitors is based on the stabilization of a cleavable complex which is normally a transient reaction intermediate (Chen and Liu, 1994; Froelich-Ammon and Osheroff, 1995). The cleaved intermediate can be either a single strand or double strand break. Drugs which are topo II inhibitors exert their effects possibly by inhibiting the rejoining step in the breakage-rejoining cycle, thus shifting the equilibrium toward a cleavable complex (Chen and Liu, 1994; Froelich-Ammon and Osheroff, 1995). The agents which inhibit topo II and stabilize cleavable complex formation can be intercalative, e.g., DOX, amsacrine (m-AMSA), mitoxantrone, or non-intercalative, e.g., VP-16, teniposide (VM-26), and isoflavone derivative genistein (Chen and Liu, 1994; Pommier et al., 1994; Watt and Hickson, 1994; Froelich-Ammon and Osheroff, 1995). Mammalian topo IIα (170 kDa) and topo IIβ (180 kDa) isozymes exhibit similar catalytic, but different biologic, activities. Whereas topo IIα is associated with cell division, topo IIβ is involved in differentiation (Chung et al., 1989; Drake et al., 1989; Woessner et al., 1989, 1990). The 170 kDa topo II isoform is encoded on chromosome 17q21-22 while the 180 kDa topo II isoform is encoded on chromosome 3p24 (Chen and Liu, 1994; Watt and Hickson, 1994). As a target for anti-cancer agents, there is more information on the interaction with the 170 kDa topo IIα protein, although a possible role for alterations in the 180 kDa topo IIβ isoform in mitoxantrone-resistant and m-AMSA-resistant HL-60 cells has been reported (Harker et al., 1991; Chen and Liu, 1994; Froelich-Ammon and Osheroff, 1995; Herzog et al., 1998). A number of *in vitro* studies using purified or recombinant topo II enzyme have addressed determinants of drug interaction with topo II (Fry et al., 1991; Chen and Liu, 1994; Pommier et al., 1994; Watt and Hickson, 1994; Froelich-Ammon and Osheroff, 1995), and in cell systems the focus has been on enzyme levels as the determinant of drug action (Fry et al., 1991; Chen and Liu, 1994; Pommier et al., 1994; Watt and Hickson, 1994; Froelich-Ammon and Osheroff, 1995). The proliferative state of tumor cells is also an important determinant of sensitivity to inhibitors of topo II, and a correlation exists between proliferation, cell cycle stage and cytotoxicity (Nelson et al., 1986; Sullivan et al., 1986; Estey et al., 1987; D’Arpa et al., 1990).

Resistance to inhibitors of topo II reported in a number of tumor model systems is also prevalent in clinically refractory tumors (Chen and Liu, 1994; Froelich-Ammon and Osheroff, 1995). Based on the evaluation of tumor models with intrinsic or acquired resistance to the topo II inhibitors, as well as cell lines selected for resistance which express decreased levels of topo II (Chen and Liu, 1994; Froelich-Ammon and Osheroff, 1995) it has been proposed that levels of topo II are an important determinant of drug sensitivity. Although the levels of enzyme are obviously critical, there are several examples wherein drug sensitivity is not correlative with the 170 kDa topo IIα protein level (Ganapathi et al., 1991a, 1996; Chen and Liu, 1994; Pommier et al., 1994; Watt and Hickson, 1994; Froelich-Ammon and Osheroff, 1995). Alternatively, altered sub-cellular distribution of the 170 and 180 kDa isoforms may also be involved with insensitivity to topo II inhibitors (Fernandes et al., 1990; Juenke and Holden, 1993; Mirski et al., 1993; Danks et al., 1994; Feldhoff et al., 1994; Zini et al., 1994). While the identification of a mutant enzyme associated with drug resistance has been reported (Takano et al., 1992; Pommier et al., 1994) an analysis of leukemic cells from patients who have relapsed from etoposide or teniposide therapy revealed that resistance does not have to be associated with mutations in the topo II gene (Danks et al., 1993). Also mutations identified in cultured cell lines were not found in the patient samples (Danks et al., 1993). Point mutations similar to those observed with topo IIα in m-AMSA-resistant cell lines (Hinds et al., 1991) has been reported in patients with small cell lung cancer treated with etoposide (Kubo et al., 1996). In addition to these mutations that have possible relevance to patient tumors refractory to therapy, several other mutations in topo IIα and β (induced or observed in drug-resistant tumor models) have been described that can confer resistance to drugs that target the enzyme (Chikamori et al., 2010). While studies with these mutant forms of topo II are informative, their functional role remains controversial, since they are generally not observed in patients with tumors that are clinically resistant to drugs that target the enzyme.

## ANTHRACYCLINES, TOPOISOMERASE IIα, AND BREAST CANCER

The recognition that topo IIα is a putative target of DOX, a clinically active anthracycline in the treatment of breast cancer, has led to several reports correlating anthracycline sensitivity with topo IIα expression. Major focus has been on human epidermal growth factor receptor 2 (HER2) and topo IIα expression based on their localization in chromosome 17 as well as determinants of sensitivity to trastuzumab and anthracyclines, respectively. Indeed several reports have established expression of topo IIα in predicting sensitivity to adjuvant anthracycline therapy (Oakman et al., 2009; Brase et al., 2010; Kawachi et al., 2010; Di Leo et al., 2011; Du et al., 2011; Nikolényi et al., 2011; O’Malley et al., 2011). The evaluation of tissue inhibitor of metalloproteinase (TIMP-1) with HER2 or topo IIα has also suggested that a HT profile (HER2 amplified and/or TIMP-1 negative) or 2T profile (topo IIα aberrant and/or TIMP-1 negative) with substantial reduction in mortality but not relapse free survival events following adjuvant anthracycline containing therapy (Ejlertsen et al., 2010; Hertel et al., 2012). Overall, while topo IIα expression is possibly a determinant of response to anthracycline containing therapy, robust assay methodology for topo IIα and well defined prospective clinical trials will establish the predictive value.

## PHOSPHORYLATION OF TOPOISOMERASE II

The proliferation and cell cycle phase dependent post-translational modification by phosphorylation of the 170 and 180 kDa topo II protein (Heck et al., 1989; Kroll and Rowe, 1991; Saijo et al., 1992; Burden et al., 1993; Burden and Sullivan, 1994; Kimura et al., 1994a, b), is also linked to increased enzyme activity and DNA cleavable complex formation (Heck et al., 1989; Kroll and Rowe, 1991; Saijo et al., 1992; Burden et al., 1993; Burden and Sullivan, 1994; Kimura et al., 1994a, b). Since phosphorylation of topo II during the cell cycle regulates activity of the enzyme (Ackerman et al., 1985; Sahyoun et al., 1986; Saijo et al., 1990; Cardenas et al., 1992, 1993; Cardenas and Gasser, 1993; Wells et al., 1994), the role of altered topo II phosphorylation in drug resistance has been studied. Takano et al. (1991) reported hyperphosphorylation of topo II in etoposide-resistant cells based on phosphorylation normalized for a 10-fold reduced enzyme level in the etoposide-resistant subline compared to parent cells. Since the phosphorylation of topo II is essential for catalytic events of unknotting and decatenation during cell replication, the observed hyperphosphorylation could represent a compensatory event for the reduced protein level. The topo IIα in these etoposide-resistant cells has a Ser861-Phe mutation, suggesting that these cells which hyperphosphorylate topo IIα, also express mutant serine residue (Kohno et al., 1995). Hypophosphorylation of topo IIα in the teniposide-resistant cells was >twofold compared to the parental cells, with serine being the primary phosphorylated amino acid in the sensitive or resistant cells (Chen and Beck, 1995). Subsequent studies by Ritke et al. (1994a), 1995 in etoposide-resistant K562 human leukemia cells have suggested that hypophosphorylation of topo IIα in these cells is due to decreased levels of the protein kinase C isoform ß_II_. Studies *in vitro* using *Drosophila melanogaster* topo II have demonstrated that phosphorylation of topo II by casein kinase (CK) II and protein kinase C can decrease drug stabilized DNA cleavable complex and increase DNA religation, suggesting that phosphorylation can confer relative drug resistance (DeVore et al., 1992). While this effect may be due to the simultaneous use of two different kinases, a role for site specific phosphorylation differences was not discussed. Phosphorylation of topo IIα by CKII has also been reported to not affect the DNA relaxing or DNA unknotting activity (Kimura et al., 1996). In contrast to these reports we have demonstrated, following metabolic labeling of cells with [^32^P]-orthophosphoric acid, the hypophosphorylation of 170 kDa topo II in the absence of any decrease in steady state topo II protein levels in three different model systems resistant to topo II inhibitors (Ganapathi et al., 1991a, b, 1996).

## FUNCTIONAL ROLE FOR INTRACELLULAR CALCIUM AND SITE-SPECIFIC PHOSPHORYLATION OF TOPOISOMERASE IIα

Potential mechanisms affecting reversibility of the drug-induced DNA cleavable complex (Hsiang and Liu, 1989) have been reported, and in resistant sublines a specific role for cleavable complex instability has been suggested (de Jong et al., 1993; Ritke et al., 1994b). The incubation of Chinese hamster DC3F cells in calcium-free medium or chelation of extracellular calcium with [ethylenebis(oxyethylenenitrilo)]tetraacetic acid (EGTA) has been reported to protect against the cytotoxicity of VP-16 (Bertrand et al., 1991). However, under these same conditions, VP-16-induced DNA single strand break frequency in calcium-depleted cells was reported to be comparable to control cells (Bertrand et al., 1991). The amount of phosphorylated topo IIα in cells is obviously a balance between kinase and phosphatase activity, and our data on hypophosphorylated topo IIα in etoposide-resistant cells may be linked to enhanced phosphatase activity. We have previously reported that okadaic acid an inhibitor of protein phosphatases 1 and 2A does not affect the cytotoxicity of the topo II inhibitor DOX (Kawamura et al., 1996b). The activity of protein phosphatase 2B (calcineurin) is enhanced following phosphorylation by calcium-calmodulin protein kinase II (Hashimoto and Soderling, 1989; Sacks et al., 1995) or suppressed by calmodulin inhibitors (Klee et al., 1988). Thus, enhanced phosphorylation of topo IIα (Kawamura et al., 1996a) in the presence of the inhibitors of calcium-calmodulin regulated processes, e.g., TFP or 1-[*N*,*O*-bis(1,5-isoquinolinesulfonyl)-*N*-methyl-L-tyrosyl]-4-phenylpiperazine (KN-62) possibly involves inhibition of calcineurin activity, which leads to potentiation of DNA cleavable complex formation and cytotoxicity of topo II inhibitors.

Based on the potentiation of DOX cytotoxicity and DNA damage by TFP and other inhibitors of calcium-calmodulin regulated cellular events we sought to determine whether intracellular calcium could be involved in affecting DNA damage induced by drugs that target topo II. Manipulating intracellular “free” calcium was achieved with the chelator (Ganapathi et al., 1996) 1,2-bis(*o*-aminophenoxy)ethane-N,N,N′,N′,-tetraacetic acid tetra(acetoxymethyl) ester (BAPTA-AM). In wild-type cells pre-treatment with BAPTA-AM followed by the topo II inhibitor etoposide (VP-16) led to significant reductions in drug stabilized DNA cleavable complex formation and cytotoxicity (Ganapathi et al., 1996). These results on reduced DNA cleavable complex formation following buffering of intracellular calcium, in general support the original observation of Osheroff and Zechiedrich who reported that in experiments with the purified enzyme *in vitro*, calcium was able to promote high levels of *D. melanogaster* topo II-mediated DNA cleavage (Osheroff, 1987; Osheroff and Zechiedrich, 1987). Also, pre-treatment of wild-type cells with BAPTA-AM led to hypophosphorylation of topo IIα (Ganapathi et al., 1996). In order to determine whether the hypophosphorylation of topo IIα was site-specific, we carried out 2D mapping with tryptic digests of immunoprecipitated topo IIα from in DOX-resistant or wild-type cells pre-treated with BAPTA-AM (Ganapathi et al., 1996; Chikamori et al., 2003). Interestingly, we found that site specific hypophosphorylation of topo IIα in DOX-resistant or wild-type cells pre-treated with BAPTA-AM was comparable (Ganapathi et al., 1996; Chikamori et al., 2003). Using liquid chromatography-tandem mass spectrometry, we identified the hypophosphorylated site as serine 1106 in topo IIα (Chikamori et al., 2003).

To establish the functional role for serine 1106 in topo IIα, mutation of serine 1106 to alanine (S1106A) was carried out and found to abrogate phosphorylation of the phosphopeptides that were found either in the DOX-resistant cells or wild-type cells treated with BAPTA-AM. Using purified wild-type or mutant (S1106A) topo IIα expressed in BJ201 cells, we observed decreased decatenation activity as well as etoposide stabilized DNA cleavable complex formation with the mutant enzyme (Chikamori et al., 2003). A functional role *in vivo* for serine 1106 in resistance to inhibitors of topo II was also established using the yeast system wherein resistance to the cytotoxic effects of etoposide and m-AMSA was observed (Chikamori et al., 2003).

Since serine 1106 is flanked by CKI consensus sequences, and phosphorylation of this site is regulated by calcium, we probed the effect of inhibitors of CKI (Grozav et al., 2009). Treatment with CKI-7 or IC261 that inhibit CKI activity, both hypophosphorylation of serine 1106 and decreased etoposide stabilized DNA cleavable complex formation was observed, suggesting a potential role for CKI phosphorylation of topo IIα (Grozav et al., 2009). In the CKI family, a functional role for calcium regulatable CKIδ and/or CKIε in phosphorylating serine 1106 and affecting drug stabilized topo II DNA cleavable complex formation was established using small interfering RNA (siRNA) that target these isozymes of CKI (Grozav et al., 2009). Although a precise role for site specific hypophosphorylation of topo IIα and resistance to inhibitors of topo II in patient tumors has not been established, in our preliminary studies with early passage cultures of acute myeloid leukemia and non-small cell lung cancer from patients, we have observed a correlation of site specific hypophosphorylation of topo IIα and decreased drug stabilized DNA cleavable complex formation and/or cytotoxicity with inhibitors that target topo II.

In addition to phosphorylation, other post-translational modifications of topo II include sumoylation and ubiquitination (Chikamori et al., 2010). Sumoylation of topo II that is induced by inhibitors targeting the enzyme also affects cellular localization (Chikamori et al., 2010). A role for ubiquitination-proteasome pathway in regulating enzyme function has also been reported (Chikamori et al., 2010). Interestingly, in human non-small cell lung carcinoma cells, proteasome inhibition with, e.g., MG-132 following treatment with etoposide leads to enhanced apoptosis and decreased arrest of cells in the G_2_+M boundary, without apparent alteration in degradation of topo II (Tabata et al., 2001). In contrast, pre-treatment with the proteasome inhibitor followed by etoposide leads to decreased apoptosis, possibly due effects on apoptotic signaling (Tabata et al., 2001). Neither, pre- or post-treatment with the proteasome inhibitor affected DNA damage induced by etoposide, suggesting that downstream events, e.g., apoptotic response may be another strategy to enhance anti-tumor activity of topo II inhibitors.

## FUTURE DIRECTIONS

In summary, it is apparent that multifactorial mechanisms govern the sensitivity of tumor cells to the DNA damaging and cytotoxic effects of clinically useful inhibitors of topo II. Much progress has been made in identifying agents and developing strategies for enhancing cellular accumulation of topo II inhibitors in tumors with the MDR phenotype. However, differences between “acquired” and “intrinsic” resistance as well as insights on mechanisms that lead to reduced activity of topo II or compromised activation of cell death pathways in tumors from patients resistant to clinically active topo II inhibitors is an underexplored area. Thus, development of targeted drugs that can activate topo II activity and cell death pathways without enhancing treatment-induced toxicity, have considerable potential in combination therapy for clinically improving the anti-tumor efficacy of topo II inhibitors.

## Conflict of Interest Statement

The authors declare that the research was conducted in the absence of any commercial or financial relationships that could be construed as a potential conflict of interest.
